# A Tablet-Based Technology for Objective Exercise Monitoring in Vestibular Rehabilitation: Mixed Methods Study

**DOI:** 10.2196/58713

**Published:** 2025-02-04

**Authors:** Brooke N Klatt, Pedram Hovareshti, Lisa S Holt, Pamela M Dunlap, Chad Zalkin, Devendra Tolani, Susan L Whitney

**Affiliations:** 1 Department of Physical Therapy, University of Pittsburgh, Pittsburgh, PA, United States; 2 BlueHalo, Rockville, MD, United States

**Keywords:** technology, rehabilitation, vestibular, physical therapy, vestibulo-ocular reflex, ocular, physiotherapy, vision, feasibility, exercises, mHealth, mobile health, app, tablet, digital health, telerehabilitation, e-health, web-based, clinical use, physiotherapist, home exercise, usability study, mobile app

## Abstract

**Background:**

A low-cost home exercise system called VestAid has been developed to assist participants during vestibulo-ocular reflex gaze stabilization exercises outside of clinic visits. The system includes a tablet-based app for the participant and a web-based portal for the physical therapist that provides data to make judgments about exercise accuracy and performance.

**Objective:**

The purpose of this study was to assess the feasibility and acceptability of VestAid in a pilot study of 10 participants (mean age 45 [SD 19] years; 6 women) with various vestibular diagnoses.

**Methods:**

All participants completed twelve 30-second horizontal vestibulo-ocular reflex exercises in a seated position (6 “easy” and 6 “hard” exercises). The exercises differed by variations in the background color, pattern, and movement. One of the exercises was repeated to assess the test-retest reliability of the measure of gaze stability accuracy and head motion compliance during the exercise. Participants rated the difficulty of the exercises (0‐10 where 0=easy, 10=difficult) and completed usability surveys.

**Results:**

Participants completed the VestAid session without adverse events. The responses from the usability survey demonstrate the acceptability of VestAid. The mean rating of the “easy” exercises was 2.7/10 (SD 1.9). The mean rating for the “difficult” exercises across participants was 4.8/10 (SD 2.1).

**Conclusions:**

The consistency of the mean ratings of the participants with the exercise classifications (“easy” and “difficult”) suggests that VestAid has clinical utility.

## Introduction

Completion of gaze stabilization, or vestibulo-ocular reflex, exercises, such as vestibulo-ocular reflex × 1 (VORx1), have been shown to improve vestibulo-ocular reflex gain and reduce dizziness [1-3]. Prescription of gaze stabilization exercises has also been linked to decreased risk of falls [4]. Current clinical practice guidelines recommend that people with vestibular disorders complete gaze stabilization exercises at a minimum of 3 times per day for a total of at least 12‐20 minutes each day if they have difficulty with vision or dizziness with head movement [5,6].

A retrospective chart review of 104 participants referred to vestibular rehabilitation postconcussion found that eye-head coordination exercises were the most common exercise prescribed by vestibular physical therapists, with the most common frequency and duration being 3 times per day for 60 seconds [7]. To achieve the recommended dose for VORx1 exercise during vestibular rehabilitation, most of the exercises must be completed outside of the clinic without expert supervision, which prevents participants from receiving timely individualized feedback.

Determining VORx1 exercise compliance is challenging for rehabilitation professionals. In the clinic, evaluation of gaze compliance and head speed compliance may vary based on clinician experience, and there is currently no easy, objective method to record VORx1 exercise performance.

**Figure 1.**
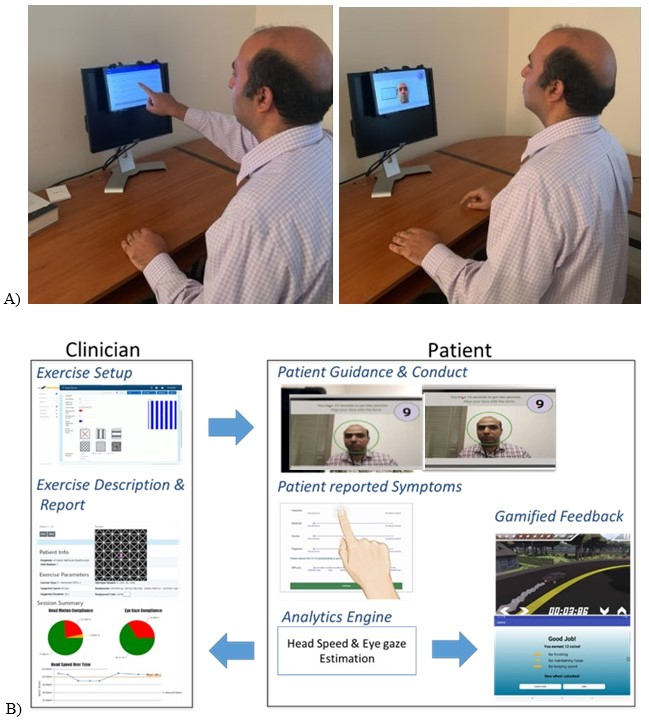
VestAid tablet (**A**) and web portal (**B**).

VestAid [8] is an innovative tablet-based app for home or clinic use to objectively track exercise compliance and provide VORx1 performance feedback to both the treating physical therapist and the participant (Figure 1). Video instruction helps participants recall how to do the VORx1 exercises and a metronome guides head speed during the exercises. With the tablet camera, facial and eye recognition software is used to analyze the accuracy of head and eye movement during the exercise. An unconstrained face detection method (where the face in the frame has arbitrary position and orientation variations) [9] combined with facial landmark detection and convolutional neural network models are used to determine the face image, refine landmark points (eye and nose positions), and capture face shape [8,10,11]. Head angles are computed using the filtered data from facial landmarks. A peak detection algorithm is used to determine head-speed compliance. The system classifies eye gaze direction as on- and off-target which is then used to determine eye-gaze compliance. The participant records their symptoms before and after each exercise which is then combined with the eye and gaze performance to provide feedback to the participant and the physical therapist.

While VestAid is thought to provide useful information to the participant and clinician, the feasibility and usability from a participant perspective is not yet known. The purpose of the study was to evaluate the usability of the VestAid technology from the perspective of the participant. Prior to using VestAid in a clinical trial, it was important for us to know whether the participants found the device easy and acceptable to use. We hypothesized that VestAid would be both feasible and acceptable to participants with dizziness who complete VORx1 exercises.

## Methods

### Participants

Ten participants receiving vestibular rehabilitation for dizziness were recruited in this single-site pilot study in a tertiary balance center. All participants were 18 y or older and were diagnosed with a vestibular disorder by a neurologist who specializes in otology. Participants were excluded if they had a baseline dizziness rating of >5 out of 10 on a verbal analog scale (with 10 being the worst imaginable dizziness), visual acuity worse than 20/40, loss of protective distal sensation, or less than 5 degrees of ankle dorsiflexion. Previous experience has shown that people with severe baseline dizziness might not be able to complete the study.

### Ethical Considerations

Informed consent was obtained from all participants and the study was approved by the University of Pittsburgh Institutional Review Board (STUDY19050389).

### VORx1 Exercise Description

Participants performed horizontal VORx1 (a yaw head movement exercise done at 80 beats per minute while focusing on a stationary target) for a total of 12 trials of 30 seconds each during the single study session. There was a 1-minute rest break between each trial and per study protocol, the next trial was not initiated until symptoms returned to baseline. The study team chose a standardized speed for this usability study and opted for a relatively slower speed that we felt all participants could complete. Twelve trials of 30 seconds provided a total of 6 minutes of gaze stabilization exercise during the study session. If the gaze stabilization exercise is completed 3 times per day as is recommended for vestibular hypofunction, the dose of 6 minutes in our study session is on target for the recommended daily dose for gaze stabilization exercises (3 times per day for 6 minutes equals 18 min of gaze stabilization).

**Figure 2.**
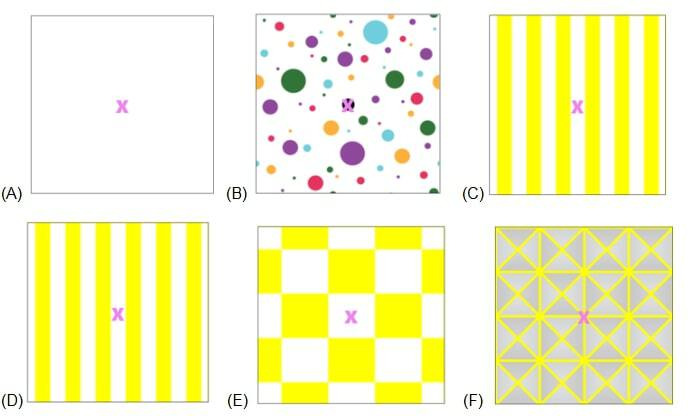
Background patterns of the easy vestibulo-ocular reflex × 1 (VORx1) exercises; background stationery for all 6 exercises.

Each exercise varied in difficulty level by manipulating the background colors, patterns, and movement of the visual scene. The study team classified 6 of the exercises as being “easy” and 6 as being “difficult.” The exercises were selected and classified as “easy” or “difficult” based on pilot testing with study team members and the clinical experience of the research team. Insights from both the pilot testing and the team’s clinical expertise informed the final categorization, supporting appropriate exercise selection for study participants. The background of the easy exercises had low color contrast with no movement whereas the more difficult exercises had higher color contrast, and all but one trial incorporated a moving background. The 6 easy exercises were completed first in a randomized order followed by a second group of 6 difficult exercises, also in randomized order. This design was planned as we were unsure if participants would tolerate the moving and busier backgrounds and we wanted to allow for adequate comparisons of exercises completed. All exercises were completed in the seated position with the tablet positioned 3 feet away from the participant. A minimum of a 1-minute rest was given between trials. The visual fixation gaze stabilization target was viewed on the tablet screen and the exercise was video-captured and recorded by the tablet. For all 12 exercises, the optotype of the target was a pink “X” with a 30-point font size. The background patterns and movement during the more difficult exercises differentiated “easy” exercises from the “difficult” (Figures2  3).

**Figure 3.**
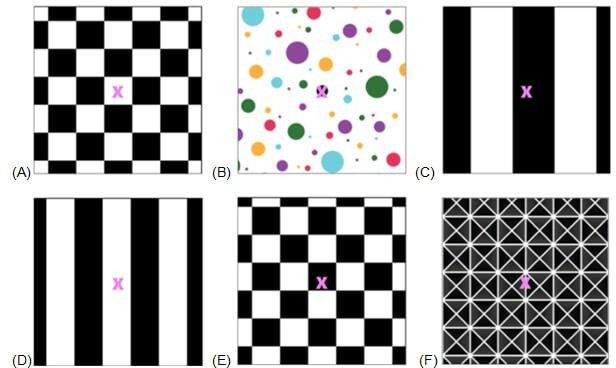
Background patterns and movements of the difficult vestibulo-ocular reflex × 1 (VORx1) exercises. Background movements are as follows: (A) stationary, (B) rotating clockwise, (C) moving left to right, (D) oscillating horizontally, (E) moving down to up, and (F) oscillating vertically.

Exercises are easily selected in the VestAid web portal for the desired parameters including exercise duration, head speed, target size, and background (type, color, stationary or moving speed, and direction of movement). The patient-facing tablet-based app provides written exercise instructions and an audible metronome to ensure proper head speed. Image-processing algorithms use facial recognition features from the camera in the tablet to track the patient’s gaze fixation, head movement amplitude, and speed. VestAid estimates key parameters, including 3D head angles (roll, pitch, and yaw) and eye movements, in near real-time using advanced machine learning algorithms. Using these data, VestAid calculates compliance metrics for head and eye movements, such as the percentage of the exercise performed at the correct speed. Additionally, the app collects patients’ pre and postexercise symptoms (dizziness, headache, nausea, and fogginess) along with their perceived difficulty of the exercise.

### Data Collection and Analysis

All participants completed the Dizziness Handicap Inventory [12] prior to the initiation of the exercises. Participants completed a 0 to 10 visual analog score rating (where 0=no symptoms and 10=worst imaginable symptoms) of their dizziness, headache, nausea, and fogginess before and after each VORx1 exercise [13]. Additionally, after each VORx1 exercise, participants rated the difficulty of the exercise on a 0 to 10 visual analog scale (VAS) (0=no difficulty and 10=most difficult) [14]. After the participants performed all 12 VORx1 exercise trials, they were asked to complete a brief usability survey with a 10-point Likert scale to note their level of agreement with 10 statements and 6 open-ended questions. The usability survey was administered on paper and asked open-ended questions such as “What did you like most in the app?” and “What did you like least in the app?” Descriptive statistics and reliability analysis were conducted using SPSS (version 28; IBM).

## Results

The 10 participants (6 females) had a mean age of 45.3 (SD 18.7) years and a mean Dizziness Handicap Inventory score of 41.4 (26.7) indicating moderate handicap due to dizziness on average (Table 1). The number of vestibular rehabilitation visits completed prior to the study session ranged from 1 to 15 (mean 6; median 5) and the vestibular diagnoses included: unilateral vestibular loss (n=4), vestibular migraine (n=3), postconcussion (n=2), and dizziness of unknown etiology (n=1).

**Table 1.** Participant demographics.

	Age (years)	Sex	Diagnosis	Vestibular physical therapy visits completed	Dizziness handicap inventory
1	31	Male	Vestibular Migraine	10	52
2	41	Male	Postconcussion	9	64
3	31	Male	Unilateral vestibular hypofunction	3	4
4	80	Male	Dizziness of unknown etiology	2	88
5	43	Female	Vestibular Migraine	1	26
6	27	Female	Postconcussion	3	28
7	70	Female	Unilateral vestibular hypofunction	7	30
8	52	Female	Vestibular Migraine	2	74
9	24	Female	Unilateral vestibular hypofunction	15	26
10	54	Female	Unilateral vestibular hypofunction	7	22

The changes in dizziness, headache, nausea, and fogginess are summarized below. There was a total of 120 exercises (10 participants each completed 12 exercises) and while symptoms often increased following the exercise trial, all participants returned to baseline within 1 minute of rest. Exercise difficulty ratings had variable distributions on the 0‐10 scale (where 0=easy and 10=difficult). The recorded gaze stabilization and head speed compliance varied among exercises and participants. Participants were not provided feedback on the objective output from VestAid related to their gaze stabilization accuracy and head speed compliance. The distribution of difficulty ratings is illustrated in Table 2.

**Table 2.** Frequency of reported exercise difficulty ratings for 120 exercises.

Reported difficulty rating (0‐10)	Frequency, n
0	12
1	10
2	19
3	17
4	19
5	14
6	15
7	11
8	5
9	1
10	0

**Figure 4.**
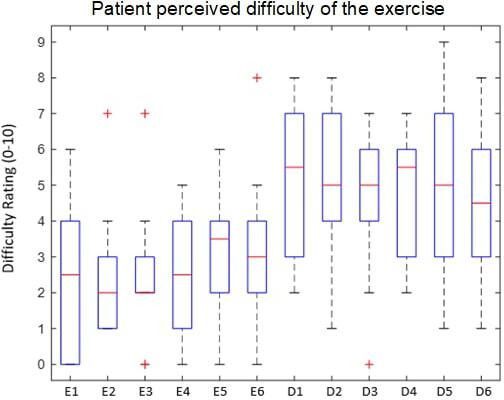
Participant perceived difficulty with the vestibulo-ocular reflex × 1 (VORx1) yaw exercises. D: difficult; E: easy

Prior to collecting data, physical therapists categorized 6 exercises as easy and 6 exercises as difficult. Figure 4 reveals that the participants rated the exercises categorized as difficult at higher difficulty ratings than those that were categorized as easy, validating the physical therapist’s determination of exercise difficulty.

As illustrated in Table 3, 100% of the participants strongly agreed that the instructions were clear and VestAid was easy to use (usability study). Neutral responses were recorded from one respondent for statements about the size and color of the target being easy to see and the ease of rating the exercise difficulty. A common theme of “easy to use” was inferred from the responses to the question about what was liked most in the app from responses such as, “self-explanatory,” “user friendly,” “quick to navigate,” and “could be used at home.” The responses regarding what was liked least about VestAid included “user interface for reporting the symptom and difficulty rating” (n=5), “visual appeal of the user interface” (n=1), “metronome speed” (n=1), and “distance of tablet too far away” (n=1).

**Table 3.** Distribution of responses (n=10) to statements provided on the VestAid usability survey.

Statement	Strongly disagree	Disagree	Neutral	Agree	Strongly agree
The exercise instructional video was easy to understand.	—^a^	—	—	—	10
The exercise steps were easy to follow.	—	—	—	—	10
The size of the exercise target (the X) made it easy to see.	—	—	1	2	7
The color of the exercise target (the X) made it easy to see	—	—	1	2	7
The metronome sound made it easy to turn my head at the right speed.	—	—	—	1	9
It was easy to rate my symptoms using the scales.	—	—	—	5	5
It was easy to rate the difficulty of the exercises.	—	—	1	3	6
Thirty seconds of exercise was easy to complete.	—	—	—	3	7
The break between exercises was enough.	—	—	—	3	7
The exercises I did using the app were similar to ones I have done before.	—	1	—	4	5

a Not applicable.

## Discussion

### Principal Findings

In this study, participants with vestibular disorders were able to complete the gaze stabilization exercise trials using VestAid without adverse events or complaints. Our sample was heterogeneous which is representative of the range of patients that are seen in vestibular rehabilitation clinics. The study sessions were tolerated by the participants without any major increases in visual analog symptom ratings. Only 7% of the exercises caused a greater than 1-point increase in the VAS for symptoms of dizziness. Of the 7% of the exercises that caused greater than a 1-point increase in dizziness, all but one were exercises from the “difficult” category and the ratings were from 3 unique participants. Only 3%, 4%, and 6% of the exercises caused an increase of greater than 1 point on the VAS for headache, fogginess, and nausea respectively. This provides us with information that displaying the gaze stabilization target and background on an electronic tablet (compared with being displayed on paper) is feasible. In this study, we chose to use an optotype that was pink as it was visible against white, black, and yellow backgrounds. We currently do not know how contrasting colors impact the difficulty of a gaze stabilization exercise and future work should explore this research question.

Overall, the feedback from the participants was positive and VestAid was well received. Participants reported that performing exercises with moving backgrounds was more difficult than backgrounds that did not move, and they indicated that increasing the speed of the background made the exercise more difficult. Participants reported that the exercises were easy to perform and that the instructions were easy to understand. We believe that the speed of head movement (80 bpm) and the completing the exercises while seated contributed to the ability of all participants being able to complete all 12 trials which was a goal of our study. It is important for new investigations to explore the optimal ways to increase the challenge and difficulty of the exercise. The participant’s perceived exercise difficulty data combined with symptom rating and the objective information about head speed and gaze stability may have clinical utility in determining when exercises should be progressed, stay the same, or be made easier in future trials or sessions. While exercise prescription was not a goal of this study, the ease of inputting symptom ratings and perceived difficulty were important for our research question examining the acceptability and usability of VestAid.

Many barriers currently exist to the completion of gaze stabilization exercises at home. Inherent to all home exercise programs, the understanding of the exercise and the motivation to complete the exercises present barriers to completion. In a systematic review, Essery et al [15] identified factors that predict whether participants will adhere to physical therapy exercise programs. They reported that self-motivation, social support, self-efficacy, and previous adherence to physical therapy exercises were positively correlated with exercise adherence in the home. Not all participants adhere to physical therapy home-based programs without social support (including supervision and encouragement from friends, family, or a physical therapist) [15].

The VestAid vestibular exercises simulate supervised exercises because they provide the physical therapist with access to performance and symptom data. Recording and tracking exercise performance makes the participant accountable to the physical therapist, and this may increase exercise adherence with VestAid. Outside of the clinic, participants do not receive feedback as to whether they are successfully completing gaze stabilization exercises (ie, maintaining visual fixation on the target and moving their head at the correct speed). Additionally, it is difficult for physical therapists to discern whether participants are completing the exercise at the appropriate level of challenge outside of clinic visits. Programs such as VestAid can make it possible for physical therapists to track participant progress and provide feedback based on performance outside of in-person sessions.

There are regions of the world where access to specialized expert care is not available [16]. VestAid may assist in the telehealth management of people with vestibular disorders, similar to other conditions that physical therapists manage via telehealth [16-23]. Internet-based vestibular rehabilitation blended with face-to-face rehabilitation and in-clinic vestibular rehabilitation were superior to usual care which was provided by a general practitioner [23]. VestAid could be used exclusively via telehealth or via a blended telehealth and face-to-face vestibular rehabilitation program.

Acceptance of technology is a consideration with people with vestibular disorders. In a systematic review [24], older adults were more adherent to technology-based programs than traditional exercise programs when provided feedback. Using VestAid, exercises can be performed sitting or standing, depending on the clinician’s judgment. There are obvious fall concerns with older adults performing balance exercises in the home unsupervised, so a clinician’s judgment is required to ensure participant safety. In a recent meta-analysis of randomized trials on the effects of exercise on fall injuries, exercising older adults showed very low injury rates, and exercise compliance was good [25]. This meta-analysis [25] appears to support the use of at-home standing or sitting balance exercises as low-risk activities in older people with vestibular disorders.

Motivation and understanding of the exercise can impact the completion of gaze stabilization exercises at home. VestAid has the capability to monitor gaze stabilization exercise performance digitally and performance can be reviewed with the participant in face-to-face interactions. In older people post stroke, remotely connecting the physical therapist and the participant via technology improved exercise adherence [26]. Technology features within VestAid might motivate and promote adherence to the prescribed home exercise program. Additionally, technology has the potential to enhance the participant’s understanding of how to complete their exercise program and should be explored in future work.

Future work should also explore the efficacy and effectiveness of using technology in vestibular rehabilitation. Clinical trials should investigate the optimal dose of gaze stabilization exercises and specific parameters that vary the challenge of the exercise [27]. Future work should also investigate the use of technology to progress or regress a customized vestibular exercise program based on symptoms, perceived difficulty, and objective performance with gaze stabilization accuracy and head motion compliance. The reliability of VestAid in recording gaze stabilization should be assessed. Additionally, the determination of acceptable scores for gaze stabilization accuracy (the ability of the eyes to stay focused on the target during VORx1 exercise) and head speed compliance (percentage of time that the head moves at the prescribed speed) need to be determined.

### Conclusion

VestAid is feasible to use in a clinical setting and acceptable to people with vestibular disorders. By monitoring exercise performance and providing near-immediate feedback, the VestAid app may improve exercise compliance and increase motivation to complete home exercises. With access to detailed performance data, clinicians can better manage participant progression between visits, thus potentially optimizing vestibular rehabilitation outcomes and speeding recovery.

## Abbreviations

VAS: visual analog scale
VORx1: vestibulo-ocular reflex × 1
